# Student-athlete dual careers in South African public universities: insights from an ecological perspective

**DOI:** 10.3389/fspor.2026.1798187

**Published:** 2026-04-17

**Authors:** Funani Mabala, Siphesihle Vundisa, Karabo Maila, Siphokazi Mbali, Natasha Janse van Rensburg

**Affiliations:** Department of Sport and Movement Studies, Faculty of Health Sciences, University of Johannesburg, Johannesburg, South Africa

**Keywords:** academic–athletic balance, dual career, ecological systems theory, South African higher education, student-athletes

## Abstract

Student-athletes must navigate the complex demands of academic work and high-performance sport, often within higher education environments that place competing pressures on their time, energy, and performance. This study explores how student-athlete experiences differ across two South African public universities that vary primarily in their geographic setting, using Bronfenbrenner's Ecological Systems Theory and the Process–Person–Context–Time model as a guiding framework. An exploratory qualitative design was employed, with semi-structured interviews conducted with 22 undergraduate and postgraduate student-athletes (9 women, 13 men) engaged in high-performance sport. Data were analysed using inductive thematic analysis. Findings reveal that academic demands constitute the most persistent source of stress, driven by externally regulated assessments and long-term academic consequences, whereas athletic pressures are largely development-focused and internally regulated. Personal attributes such as motivation, resilience, and goal orientation, along with peer and team support, emerged as critical resources; however, injuries, transitions, and cumulative fatigue frequently disrupted coping mechanisms. Despite differences in institutional context, experiences were broadly similar, highlighting that dual-career engagement is shaped by ongoing interactions between individuals and their environments rather than singular determinants. Based on these insights, a conceptual framework is proposed that integrates person, process, context, and time dimensions, offering both theoretical and practical guidance for designing ecologically informed support systems that enhance sustainable dual-career development in higher education.

## 1 Introduction

Student-athletes navigate numerous challenges in balancing the demands of academics and high-performance sport (1). As dual-career individuals, defined as full-time or part-time students enrolled at a university and engaged in organised and competitive sports (2), they must manage the simultaneous pressures of academic success and athletic achievement, which can impact their overall well-being (3–5). Evidence indicates that these competing priorities create both internal and external pressures, often leading to increased stress, anxiety, and poorer academic results (6–8). Considering these challenges, structured and contextually responsive support systems are essential to enable student-athletes to manage their dual responsibilities more effectively.

Within higher education institutions (HEIs), dual careers require continuous negotiation of time, energy, and identity across academic and sporting domains (9). Although the student-athlete dual-career construct is well established, much of the extant literature has approached these demands primarily from an individual-level perspective, emphasising attributes such as time management, motivation, coping skills, and resilience (10, 11). While such factors are undeniably important, this focus risks underplaying the broader structural and relational contexts that shape student-athletes’ experiences. Empirical studies demonstrate that balancing academic workloads with high-performance sport commitments frequently produces role conflict, competing expectations, and chronic time pressure, often resulting in delayed academic progression, emotional fatigue, and sustained stress (12, 13). However, these pressures are not experienced uniformly; rather, they are mediated by the environments in which student-athletes are embedded (14).

## 2 The current study

Recent scholarship has increasingly recognised that dual-career experiences cannot be fully understood without attention to the institutional and social contexts in which student-athletes operate (14–16). Institutional practices, including assessment scheduling, attendance policies, and flexibility from academic staff, can either mitigate or exacerbate role conflict (17). Similarly, expectations from coaches, lecturers, and peers shape how student-athletes prioritise their roles and interpret success and failure (4, 18). Despite this growing recognition of contextual influence, there remains limited empirical research that systematically examines how differing higher education environments shape student-athletes’ lived experiences, particularly within Global South contexts. Guided by Bronfenbrenner's bioecological Process–Person–Context–Time (PPCT) framework, this study examines how university student-athletes navigate the dual demands of academic and athletic participation. By exploring how personal characteristics, relational processes, institutional environments, and temporal pressures interact, the study aims to provide a more holistic understanding of how dual-career experiences are shaped and managed within university sport contexts.

Accordingly, the study addresses the following research questions:

1. How do student-athletes experience and manage dual-career demands across person, process, context, and time dimensions?
2. How do institutional structures and relational dynamics shape these experiences?
3. How do temporal pressures and critical periods influence adaptation and stress?

### 2.1 Applying the PPCT model to student-athlete dual careers

Bronfenbrenner's bioecological theory, operationalised through the PPCT model (19), offers a comprehensive framework for examining the complexity of student-athletes’ dual careers. Rather than locating challenges solely within the individual, the PPCT model emphasises the dynamic interplay between personal characteristics, everyday interactions, institutional and sociocultural environments, and development over time. Recent work by Brandão et al. (20) has been particularly influential in translating the PPCT model into applied sport contexts, demonstrating how the framework can be operationalised to understand athlete development and lived experience. Within this framework, the *Person* component captures individual attributes such as motivation, coping strategies, emotional regulation, and self-perceptions of competence, all of which influence how student-athletes respond to dual-career demands (19). Research indicates that high motivation and strong athletic identity may support persistence but can also intensify pressure and self-expectations, particularly when academic demands conflict with sporting priorities (21, 22).

The *Process* component foregrounds proximal interactions that occur in student-athletes’ daily lives, including relationships with coaches, lecturers, teammates, and classmates. These interactions are central to how dual careers are negotiated in practice. Supportive coach–athlete and lecturer–student relationships have been shown to buffer stress and enhance perceived balance, while misaligned expectations or poor communication can heighten conflict and emotional strain (23, 24). The *Context* component is particularly salient in HE settings, where institutional structures, resources, cultures and climates vary considerably (25, 26). Universities differ in their approach to supporting student-athletes, ranging from highly structured dual-career systems to more fragmented or informal arrangements (27). Internationally, coordinated support models have been documented in contexts such as the United States and parts of Europe and Asia (28), yet there is limited evidence on how institutional context shapes student-athlete experiences in South African HEIs. Finally, the *Time* component (chronosystem) draws attention to how dual-career experiences evolve across academic years and athletic seasons. Pressures often accumulate over time, particularly during periods of transition such as progression to postgraduate study, increased competitive demands, injury, or approaching graduation (29). Understanding these temporal dynamics is essential for capturing the longitudinal nature of student-athlete development and well-being.

While there is an increasing call for more student-centred approaches to supporting student-athletes (30), it is not always feasible for individual institutions to conduct comprehensive empirical investigations of their own dual-career systems. The PPCT model offers a theoretically grounded framework to examine how student-athlete experiences emerge through interactions between personal characteristics, daily relational processes, institutional structures, and temporal pressures. Guided by this framework, the present study explores the lived experiences of student-athletes across two South African public universities with differing contextual features. Rather than serving as a strict comparative design, the inclusion of two institutions enhances contextual diversity and enables examination of how dual-career processes unfold within varied higher education environments. By adopting an ecologically grounded lens, this study seeks to illuminate how structural conditions, interpersonal dynamics, and individual dispositions converge to shape student-athletes’ lived experiences within the Global South (20).

## 3 Methods

### 3.1 Design and procedure

This study adopted an exploratory qualitative design rooted in narrative inquiry and guided by a postmodernist, interpretivist paradigm, aiming to gain an in-depth understanding of the lived experiences of student-athletes balancing dual academic and sporting commitments. The interpretivist lens was chosen to explore how meaning is co-constructed between participants and researchers, recognising that experiences are shaped by social, cultural, and institutional contexts. Reflexivity was central to the research process. The research team, with professional experience in sport management and higher education, acknowledged that their backgrounds could influence data collection and interpretation. Reflexive strategies, including journal, peer debriefing, and careful documentation of analytic decisions, were employed to ensure that participants’ voices remained central to the analysis.

Ethical approval was obtained from the research ethics committees of both HEIs, and gatekeeper permission was secured prior to data collection (REC-3653-2025 and NWU-00178-25-S1). Participation was voluntary, with informed consent obtained from all participants, separating participation and recording consent. Confidentiality was maintained through the use of pseudonyms, secure data storage, and assurances of the right to withdraw at any stage without penalty. All data were processed and stored in accordance with the Protection of Personal Information Act 2013 (Act No. 4 of 2013), known as POPIA, ensuring that personal information was collected, managed, and safeguarded in compliance with national data protection requirements. The study was conducted at two South African public universities: the University of Johannesburg (UJ), located in the metropolitan city of Johannesburg, and North-West University (NWU), Potchefstroom campus, situated in a smaller and more geographically contained setting. Both institutions are well established in high-performance sport and provide structured academic programmes. While their broader environmental contexts differ in scale and spatial organisation, each offers a context within which student-athletes negotiate academic, athletic, and social commitments.

### 3.2 Participants

A snowball sampling strategy was employed to recruit 22 (*N* = 22) student-athletes who met the inclusion criteria. Participants were eligible for inclusion if they were currently registered as full-time contact students (undergraduate or postgraduate) at the two institutions during the 2025 academic year. Eligible participants were recipients of a sports bursary awarded by the respective university sports bureau and actively engaged in semi-professional sport competitions. Participants had to be aged 18 years or older, have completed at least one full academic semester while managing both academic and athletic responsibilities, and be willing to provide informed consent and participate in a semi-structured interview. Initial participants referred other eligible student-athletes who then contacted the researcher directly to confirm their willingness to participate. Initial participants were asked to refer other eligible student-athletes, who then contacted the researcher directly to confirm their willingness to participate. Contextual information, including gender, sport code, and level of study, was collected during the interviews to enhance transferability and provide descriptive depth. Individual participant characteristics are presented in Table 1, and aggregated sample characteristics are summarised in Table 2. To improve analytic transparency while preserving confidentiality, participants were assigned structured pseudonyms that reflect selected contextual characteristics. Each pseudonym includes participant number, university affiliation, gender, and sport code (e.g., P1-UJ-M-Fo). This format enables readers to situate quotations within relevant contextual parameters without compromising anonymity. These identifiers are used consistently throughout the Results section.

**Table 1:** Individual characteristics.

ID	University	Sex	Sport	Level of study
P1-UJ-F-At	UJ	Female	Athletics	Undergraduate
P2-UJ-F-At	UJ	Female	Athletics	Postgraduate
P3-UJ-F-Ru	UJ	Female	Rugby	Postgraduate
P4-UJ-M-Ru	UJ	Male	Rugby	Undergraduate
P5-UJ-M-Ru	UJ	Male	Rugby	Undergraduate
P6-UJ-M-Ru	UJ	Male	Rugby	Undergraduate
P7-UJ-M-Ru	UJ	Male	Rugby	Undergraduate
P8-UJ-M-At	UJ	Male	Athletics	Postgraduate
P9-UJ-M-Ho	UJ	Male	Hockey	Undergraduate
P10-UJ-M-Ru	UJ	Male	Rugby	Undergraduate
P11-UJ-M-Ru	UJ	Male	Football	Postgraduate
P12-NWU-F-Ho	NWU	Female	Hockey	Postgraduate
P13-NWU-F-Ho	NWU	Female	Hockey	Undergraduate
P14-NWU-F-Ho	NWU	Female	Hockey	Undergraduate
P15-NWU-M-Ho	NWU	Male	Hockey	Postgraduate
P16-NWU-F-Ho	NWU	Female	Hockey	Undergraduate
P17-NWU-F-Ba	NWU	Female	Basketball	Undergraduate
P18-NWU-F-Ne	NWU	Female	Netball	Undergraduate
P19-NWU-M-Sq	NWU	Male	Squash	Postgraduate
P20-NWU-M-Cr	NWU	Male	Cricket	Postgraduate
P21-NWU-M-Cr	NWU	Male	Cricket	Undergraduate
P22-NWU-M-Cr	NWU	Male	Cricket	Undergraduate

**Table 2:** Aggregated participant characteristics (*N* = 22).

Characteristic	Category	*n*	%
Total participants		22	100
Sex	Female	9	40.9
	Male	13	59.1
Level of study	Undergraduate	14	63.6
	Postgraduate	8	36.4
Sport type	Rugby	6	27.3
	Hockey	6	27.3
	Cricket	3	13.6
	Athletics	3	13.6
	Football	1	4.5
	Basketball	1	4.5
	Netball	1	4.5
	Squash	1	4.5

### 3.3 Interviews

Semi-structured, face-to-face interviews were conducted at private locations on participants’ campuses, such as offices or reserved rooms, to encourage openness and ensure confidentiality. The interview guide was informed by the literature on student-athlete dual careers (9, 21) and structured around the PPCT framework (19), covering personal, relational, institutional, and temporal dimensions of dual-career experiences. The interviews focused on five key areas, each with follow-up prompts to elicit detailed accounts:

1. Daily experiences and role management: Participants described a typical day as a student-athlete, explaining how they managed academic and athletic responsibilities, and recounting instances where these roles conflicted. Institutional support mechanisms, such as scheduling accommodations or sport-specific resources, were also explored.
2. Personal challenges and coping: Participants reflected on emotional and mental challenges encountered in balancing their dual roles, including moments of fatigue, stress, or self-doubt. Follow-up prompts explored contributing factors, coping strategies, motivation, and persistence in maintaining performance across both domains.
3. Perceived pressures from others: The interviews examined the influence of key stakeholders, coaches, lecturers, teammates, and peers, on participants’ experiences, including sources of pressure, the relative intensity of academic vs. athletic demands, and the impact on well-being and performance.
4. Institutional environment and support: Participants described how their universities facilitated or constrained dual-role engagement, including perceptions of academic policies, exam scheduling, facilities, and flexibility from academic staff. Suggestions for institutional changes to better support student-athletes were also solicited.
5. Reflections on rewards, challenges, and learning: Participants discussed the most meaningful and difficult experiences in their dual careers, the factors shaping these experiences, and the lessons learned about themselves and their student-athlete role.

Each interview lasted 30–45 min, was audio-recorded with informed consent, and transcribed verbatim. Field notes captured contextual observations, non-verbal cues, and emerging reflections. Transcripts were returned to participants for member checking to verify accuracy and enhance credibility.

## 4 Data analysis

Data were analysed using reflexive thematic analysis (31), following a recursive and reflective process to identify patterns of shared meaning underpinned by central concepts. The analysis followed Braun and Clarke's six-phase process: familiarisation, coding, theme generation, reviewing, defining and naming themes, and reporting. Codes and themes were generated inductively, allowing for a nuanced understanding of student-athletes’ experiences. Reflexive memoing and peer discussion were used throughout the process to ensure interpretations remained grounded in participants’ accounts. Selected transcripts were co-coded by a second researcher, and discrepancies were discussed until consensus was reached, enhancing dependability.

### 4.1 Trustworthiness

Trustworthiness was established through multiple strategies (32). Credibility was supported through member checking, reflexive engagement with data, and repeated reading of transcripts. Dependability was ensured through the maintenance of a detailed audit trail documenting methodological decisions, coding processes, and theme development. Confirmability was addressed through reflexive journaling, peer debriefing, and explicit acknowledgement of the researcher's positionality, ensuring that interpretations were grounded in participant data. Transferability was enhanced through detailed contextual descriptions of the HEIs, participant characteristics, and dual-career demands faced by student-athletes. While the sample size was limited to 22 participants, the study prioritised depth and contextual understanding over breadth, providing rich, exploratory insights into the lived experiences of student-athletes navigating academic and sporting commitments in diverse institutional settings.

## 5 Results and discussion

This section presents the findings in an integrated results and discussion format, consistent with reflexive thematic analysis and the interpretive application of Bronfenbrenner's PPCT framework. Four interrelated themes were generated. Collectively, these themes demonstrate that the student-athlete experience is shaped by the dynamic interaction between personal characteristics, recurring relational processes, structural environments, and temporal intensification. Table 3 summarises the themes and subthemes with illustrative quotations. Each theme is presented below with integrated interpretation and theoretical positioning.

**Table 3:** Summary of themes and subthemes with illustrative quotations.

Theme	Subtheme	Illustrative quotations
1. Motivation and personal drive as engine of dual-career engagement	Internalised motivation	“That discipline, I can give myself a hand, because that comes from me. Nobody needs to hold me accountable. I hold myself accountable. And the thing is, I got… [a] very big conscious. So, if I know I didn't practice today… I wouldn't sleep at night, that's how I am…. No one had to force it on me…Sometimes it's hard to keep it up, but eventually the pressures and everything is on you, you just need to do it.” (P1-UJ-F-At).
	Self-regulation and coping	“I’m a huge planner… I block time for rest and study.” (P1-UJ-F-At); “It’s okay to cry, to talk to someone… having someone there to support you is really important.” (P6-UJ-M-Ru)
	Psychological and emotional strain	“pressures… to maintain the standard that I’ve set for myself” (P3-UJ-F-Ru); “sometimes you have those days… where I don’t think this is my calling… but at the end of the day, you get that hunger again” (P4-UJ-M-Ru)
	Identity negotiation	“The most rewarding is realising that you are able to still perform academically and still be a top athlete.” (P9-UJ-M-Ho); “It's been like three years playing basketball… I was able to improve and do things I didn't think I would do in such a short period of time.” (P17-NWU-F-Ba)
2. Proximal processes: the everyday labour of holding it together	Daily routines and temporal compression	“*class schedules, gym, field sessions, physiotherapy, family responsibilities… never a typical day”* (P1-UJ-F-At);*“its stressful but necessary”* (P8-UJ-F-Ho).
	Coaches as buffers or stress amplifiers	“[My coach] understands if I miss a session for an assignment.” (P102-UJ-F-At); “Sometimes the coach expects me to be there no matter what… it feels like academics don't matter.” (P103-UJ-F-Ru)
	Lecturers and self-advocacy	“Lecturers differ from lecture to lecture… some understand, but others don't really see sport as a reason.” (P204-NWU-M-Ru)
	Peer systems as emotional regulators	“My teammates understand the stress… we motivate each other, it really helps.” (P103-UJ-F-At); “If I miss class, someone will send me the work or explain what I missed.” (P203-NWU-M-Ru)
3. Institutional structures: support that exists, but does not always hold	Structural support as buffer	“That support makes everything more manageable.” (P102-UJ-F-At); “Everything we need” (P208-UJ-F-At)
	Conditionality and policy-practice gap	“Support exists, but interpretation matters…” (P101-UJ-F-At); “Don't help me besides me having to text or email.” (P107-UJ-F-Ru)
	Temporal rigidity and the transfer of regulatory burden	“The schedules are in place, and we just work around it.” (P105-UJ-F-Ru); “I'm always going to have to be on my toes.” (P108-UJ-F-At)
	Resource availability vs. utilisation	“Impossible for you to fail if you use all the resources at your disposal.” (P110-UJ-F-At); “There is support… but for me, none.” (P106-UJ-F-Ru)
4. Time, transition, and the slow accumulation of strain and growth	Adaptation over time	“First year was hard… there was a lot to adjust to.” (P21-NWU-M-Cr); “Now I plan my week… there's less stress because I know what to expect.” (P22-NWU-M-Cr)
	Critical periods and temporal intensification	“[Training] camp fell during final exams… collapsing recovery time and amplifying cognitive load.” (P3-UJ-F-Ru); “For two weeks before USSA, we train six hours a day.” (P15-NWU-M-Ho)
	Cumulative fatigue and long-term strain	“Fatigue. Being tired… each and every single day… brain is overstimulated.” (P7-UJ-M-Ru); “Catch up, catch up, catch up.” (P14-NWU-F-Ho)
	Developmental consolidation and reflective growth over time	“Not just grow[n] as a player, but as a person… shaping me for the past four years.” (P18-NWU-F-Ne); “Learning how to respond to pressure” (P2-UJ-F-At)

### 5.1 Theme 1: motivation and personal drive as engine of dual-career engagement

Motivation emerged not simply as a personal characteristic, but as the central organising force structuring student-athletes’ daily lives. Across both institutions, participants did not describe motivation as fluctuating or externally imposed; rather, it appeared deeply internalised, identity-bound, and morally regulated. Within Bronfenbrenner's PPCT framework, this reflects a powerful *Person* characteristic that consistently shaped proximal processes, how participants trained, studied, rested, and evaluated themselves.

#### 5.1.1 Internalised motivation

Within the PPCT model, the *Person* captures the individual characteristics that student-athletes bring into their dual-career environments. Participants described deeply internalised high-performance orientations that functioned as both enablers of achievement and sources of psychological strain. Motivation, internal standards, coping strategies, and identity negotiation formed an interconnected regulatory system. Several participants articulated motivation in terms of uncompromising internal accountability. One athlete emphasised that discipline was entirely self-driven, noting that “*nobody needs to hold me accountable… I hold myself accountable”* and that if training was missed they *“wouldn’t sleep at night”* (P1-UJ-F-At). This account suggests motivation operating not only cognitively but also affectively and physiologically, with accountability fully internalised rather than externally enforced. While such regulation reflects forms of autonomous motivation (33), it also illustrates how performance standards become intertwined with self-worth. Within PPCT terms, a strong *Person* characteristic intensifies the frequency and emotional weight of proximal processes (daily practice, study routines), amplifying both productivity and pressure. A similar intensity is evident in the competitive drive “*to win. I want to win. I have to win. So, yeah. Until I win, I'm going to wake up every morning and try.”* (P8-UJ-M-At). The repetition of “*wake up every morning*” conveys ritualised persistence. Motivation is embedded in daily temporality rather than tied to isolated competitions. Winning is not episodic; it structures existence. Here, *Person × Time* interaction becomes visible. The internal drive (*Person*) is reproduced across recurring daily cycles (Time), sustaining engagement even when fatigue accumulates. However, this daily moral obligation to “*try”* may also restrict psychological recovery, revealing how temporally embedded motivation can both stabilise commitment and heighten strain (34). Confidence also emerged as developmentally constructed: “*I've never questioned my ability to succeed in both because I've done it before and I succeeded in it before”* (P2-UJ-F-At). This reflects how prior mastery experiences strengthen present efficacy. Motivation here is cumulative, built across time through successful dual-role navigation. Within the chronosystem, earlier experiences shape current Person characteristics, stabilising identity integration and reinforcing belief in dual-career competence. Yet motivation was not exclusively self-referential. For several NWU participants, drive was relationally reinforced: “*My father motivates me to study… if cricket doesn’t work out, I still need to be ready for life”* (P14-NWU-F-Cr).

Here, motivation is situated within the family microsystem. Academic effort is framed as long-term security. This represents a *Person × Context* interaction, where relational expectations and future orientation strengthen regulatory commitment. The dual-career pathway is legitimised not only through personal ambition but through intergenerational accountability. Participants recognised that achieving “*100% from both is very difficult”* (P16-NWU-M-Ru). This acknowledgement introduces tension: motivation drives maximal investment, yet ecological limits constrain output. The desire for excellence exceeds available temporal and environmental capacity. Indeed, the intensity of internal drive often carried psychological cost. The psychological cost of this intensity was palpable. One participant reflected:

Just the pressure to always be on top of things. It's the pressure to always, not perfection, but very close to perfection because there's, I feel like as a student athlete, there's little room for error in terms of the mistakes you can make because there's a lot of, just a lot of expectations from a lot of people. So you don't really get the chance to fail, to make mistakes because they're so costly in terms of your academics and your sports… I think it is just the way I grew up, it just made me put a lot of pressure on myself to always be on top of things, be perfect. So yeah, I think I'm the one who puts the most pressure on myself.” (P5-UJ-M-Ru)

The absolutist language, *“always,” “perfect”, “little room for error”,* signals rigid internal standards. Within the PPCT framework, this *Person* characteristic magnifies stress when proximal processes (training sessions, assessments, recovery demands) cannot realistically meet perfectionistic expectations. Importantly, this participant locates the origins of pressure within upbringing, demonstrating how earlier contextual experiences shape current regulatory tendencies, another chronosystem influence. These internally driven standards often interacted with institutional demands to heighten strain. At NWU, participants described particularly compressed daily schedules in which academic and sporting expectations accumulated within the same day. One athlete described “*a full day of class… two tests… hockey in the afternoon and fitness also,”* explaining that the schedule left them “*mentally drained”* despite performing well academically and athletically (P12-NWU-F-Ho). Here, the interaction between strong personal standards (*Person*) and intensified structural demands (*Context × Time*) produced cumulative fatigue. Yet moments of success, such as performing well in tests or winning a match, temporarily restored meaning, reinforcing the sense that the effort was “*worth it.”* Achievement therefore functioned as psychological validation that sustained continued engagement despite exhaustion. Strain in this sense emerged not from low motivation, but from an ecological incongruence between high ambition and limited structural recovery capacity. Importantly, participants also described deliberate strategies to sustain motivation under these pressures. Planning, reflection, prayer, and positive self-talk were frequently mentioned as ways of maintaining focus. One participant, for instance, described relying on faith and self-belief to remain motivated during demanding periods (P11-UJ-M-Ru). These accounts demonstrate how *Person* characteristics actively shape proximal processes in adaptive ways, rather than operating as fixed individual traits.

#### 5.1.2 Self-regulation and coping

Self-regulation did not emerge as a separate skill set, but as an adaptive extension of motivation itself. If motivation functioned as the internal engine of engagement, self-regulation represented the mechanism through which that engine was stabilised under pressure. Within the *Person* dimension of the PPCT framework, self-regulation operated as a generative characteristic, actively structuring proximal processes across academic and athletic domains. Participants consistently described deliberate behavioural systems: meticulous planning, prioritisation, and the protection of recovery periods. One participant identified herself as “*a huge planner*” who intentionally blocked time for rest and study (P1-UJ-F-At), while another acknowledged the personal cost of these systems, stating that his “*social life takes the sacrifice… I don't really get time to see my friends, family or my girlfriend”* (P10-UJ-M-Ru). These accounts illustrate *Person × Process* interaction in action. Internal standards (*Person*) were translated into repeated daily routines (*Process*), but often through trade-offs that narrowed social and recovery spaces. Regulation therefore preserved performance, yet simultaneously intensified lifestyle restriction.

Coping strategies were also described as developmental rather than static. Participants reflected on learning, over time, how to manage emotional overload and mental fatigue. One explained that “*it's okay to cry, to talk to someone… having someone there to support you is really important”* (P6-UJ-M-Ru), while another described leaning on “*faith… and positive self-talk”* during high-demand periods (P11-UJ-M-Ru). These strategies demonstrate how proximal processes extend beyond task management to include emotional recalibration. Person characteristics were not fixed traits; they were refined through reflection, relational engagement, and spiritual grounding across repeated cycles of stress and recovery. Support systems further illustrate *Person × Context* dynamics. Peers, family members, teammates, and faith communities functioned as stabilising microsystems that buffered the intensity of internal standards (P2-UJ-F-At; P7-UJ-M-Ru; P21-NWU-M-Cr; P22-NWU-M-Cr). Through recurring supportive interactions, motivation was sustained rather than depleted. Importantly, these coping mechanisms were not emergency responses to crisis; they were embedded within daily life, reproduced across time, and strengthened through experience. These findings align with prior research showing that dual-career student-athletes rely on multi-faceted coping strategies, encompassing practical time management, psychological resilience, and social or spiritual support, to navigate the complexities of their academic and athletic commitments (15, 16, 20).

#### 5.1.3 Psychological and emotional strain

Psychological and emotional strain emerged as a significant personal and environmental challenge shaping student-athletes’ dual-career experiences. Despite strong commitment to both sport and academics, participants frequently described mental fatigue, emotional exhaustion, and periods of feeling overwhelmed. These experiences reveal the demanding psychological conditions embedded within dual-career pathways, where sustained effort across multiple performance domains places continuous pressure on personal resources.

Within the PPCT framework, psychological strain can be understood as arising from the interaction between personal motivation and the structural demands of the dual-career environment. At the *Person* level, participants demonstrated strong internalised achievement standards and a deep commitment to success in both academic and athletic domains. While these characteristics supported persistence and performance, they also generated psychological pressure when individuals felt compelled to consistently meet the high expectations they had set for themselves. One participant described this internal tension when reflecting on the “*pressures… to maintain the standard that I've set for myself”* (P3-UJ-F-Ru), illustrating how personal ambition could simultaneously function as both a motivational driver and a source of stress (35). Participants also described fluctuations in motivation during periods of fatigue and self-doubt. One athlete reflected that “*sometimes you have those days… where I don't think this is my calling… but at the end of the day, you get that hunger again”* (P4-UJ-M-Ru). These experiences highlight the dynamic regulation of motivation over time, where moments of exhaustion and uncertainty were often followed by renewed determination to continue pursuing dual goals (33). Rather than indicating disengagement, such cycles reflect the emotional labour associated with sustaining high levels of commitment within demanding developmental contexts (36, 37). Importantly, participants’ experiences demonstrate how psychological strain emerges not solely from individual characteristics but from the interaction between *Person, Process,* and *Context* (38). Sustained engagement in intensive training schedules, academic workloads, and competitive performance environments required continuous effort and self-regulation (39). When these proximal processes occurred within highly structured institutional contexts characterised by rigid timetables, performance expectations, and limited recovery opportunities, the cumulative demands often resulted in mental fatigue and emotional depletion. Similar patterns have been identified in dual-career research, where strong achievement motivation can increase vulnerability to stress and burnout when environmental demands exceed available psychological and physical resources (40, 41).

Over time, the chronosystem also played an important role in shaping these experiences. Participants described how repeated exposure to demanding academic and athletic schedules produced cycles of pressure, fatigue, and recovery across semesters and competitive seasons. Within this temporal dimension, motivation functioned both as a stabilising force sustaining continued engagement and as a potential source of strain when individuals felt unable to meet the expectations associated with their dual roles. Psychological strain therefore reflects the complex interplay between motivation, environmental demands, and temporal pressures within the ecology of dual-career sport and education.

#### 5.1.4 Identity negotiation

Identity negotiation emerged as an equally central regulatory process, through which student-athletes made sense of their dual commitments. Within the *Person* dimension, identity functioned not merely as self-description, but as an organising structure guiding effort, meaning-making, and resilience. In dual-career environments, identity integration influences how individuals allocate effort, interpret challenges, and sustain engagement across multiple performance domains (41, 42). Participants frequently described the tension inherent in occupying two demanding roles. One reflected on the challenge of being a “*student* vs. *athlete… it takes a special person”* (P1-UJ-F-At), while another explained that it was “*really tough”* when effort appeared unevenly distributed between academic and sporting responsibilities (P9-UJ-M-Ho). These accounts illustrate identity not as stable equilibrium but as continuous calibration, as academic responsibilities and athletic ambitions compete for limited temporal, cognitive, and emotional resources.

Where integration was achieved, however, it strengthened agency and self-efficacy. One participant explained that she had “*learned that I cannot be overcome by anything… I can perform exceptionally as an athlete without neglecting my academics… that has really helped me in understanding myself”* (P2-UJ-F-At). Another described the reward of dual competence, noting that “*the most rewarding is realising that you are able to still perform academically and still be a top athlete”* (P9-UJ-M-Ho). These narratives illustrate how repeated engagement in dual-role demands can consolidate identity through *Person × Process* interactions, reinforcing competence, persistence, and intrinsic motivation (33). Research on dual-career athletes similarly shows that integrated academic and athletic identities function as protective resources, strengthening resilience and promoting sustained engagement across domains (42, 43).

Identity negotiation was also shaped by structural conditions. Institutional expectations, scholarship requirements, performance metrics, and competitive pressures influenced how participants prioritised and interpreted their roles (P13-NWU-F-Ho; P19-NWU-M-Sq). This demonstrates a clear *Person × Context* interaction, as identity development occurred within, and sometimes in tension with, organisational structures that regulate performance expectations. Previous research highlights that institutional environments can either facilitate or constrain identity integration depending on the degree of flexibility, support structures, and recognition afforded to dual-career athletes (42, 44). For some participants, identity development was closely tied to experiences of transition and personal growth. One athlete described questioning her place after switching from sprinting to basketball, recalling uncertainty about whether she was “*worthy enough to play basketball.”* Over time, improvement and recognition as a “*most improved athlete”* reinforced her belief that she was capable of succeeding as a student-athlete (P17-NWU-F-Ba). Such experiences illustrate how mastery experiences contribute to identity consolidation by reinforcing competence beliefs and perceived capability within demanding developmental contexts (33, 73).

Crucially, participants emphasised that identity integration was not a definitive achievement but an iterative and developmental process. Identity was refined across academic years, shaped by prior successes and setbacks, and recalibrated in response to evolving academic and athletic demands. In PPCT terms, the chronosystem is particularly relevant, as identity coherence develops through accumulated experiences, reflective practice, and repeated exposure to dual-role challenges over time. Longitudinal research on athlete development similarly emphasises that identity formation within sport–education pathways evolves progressively as individuals negotiate transitions, performance expectations, and changing life circumstances (41, 44). Over time, this iterative negotiation enables student-athletes to stabilise a coherent sense of self capable of sustaining motivation, resilience, and commitment within the complex ecology of dual-career sport and education.

### 5.2 Theme 2: proximal processes; the everyday labour of holding it together

If motivation was the internal engine of dual-career engagement, proximal processes were the daily machinery through which that engine was tested. Within Bronfenbrenner's framework, development occurs through repeated, reciprocal interactions between the individual and their environment. In this study, those interactions were neither abstract nor occasional, they were relentless, time-sensitive, and structurally complex.

#### 5.2.1 Daily routines and temporal compression

Participants consistently described their daily routines as fragmented and compressed. One participant reflected that balancing responsibilities was complex, noting that their day involved “*class schedules, gym, field sessions, physiotherapy, family responsibilities… never a typical day”* (P1-UJ-F-At). Others described the stress of making up missed classes due to training, acknowledging that “*it's stressful but necessary”* (P8-UJ-F-Ho). These phrases, “*never a typical day” and “stressful”,* reflects instability rather than balance. Dual-career life was not a steady rhythm but an ongoing recalibration. In PPCT terms, the frequency and unpredictability of proximal processes increased regulatory demand. The more variable the microsystem interactions (lectures, training, travel), the more effortful self-regulation became. Temporal compression intensified this strain. A participant recalled that because of athletic commitments she “*… wrote all five exams in one week.”* (P3-UJ-F-Ru). This illustrates a clear *Process  ×  Time* interaction. The convergence of academic and athletic peaks magnified pressure. Importantly, the strain did not arise because either domain lacked importance, it arose because both demanded full presence simultaneously. The ecological systems were not coordinated. Another participant captured the disorganisation that followed such overload stating when asked how times such as these are managed as “*I manage them badly… it's all over the place.”* (P6-UJ-F-Ru). This admission disrupts any simplistic narrative of resilience. Not all regulatory attempts were successful. Here, proximal processes became chaotic rather than developmental, revealing how high-intensity engagement can destabilise functioning when environmental alignment is weak. They are consistent with research indicating that time-management strategies constitute a central proximal process enabling student-athletes to sustain performance and well-being across domains (45). Scheduling constraints during peak competition periods produced cumulative fatigue and required constant prioritisation. While a seemingly normal day could be viewed as mentally draining (P12-NWU-F-Ho), others highlighted the pressure of limited training windows: “*When you go to a practice you know it's only this one that you have… when it's gone, it's gone”* (P16-NWU-M-Ru). Effective scheduling not only supports academic and athletic achievement but also fosters a sense of control, agency, and resilience in the face of competing demands (21, 24).

#### 5.2.2 Coaches as amplifiers or buffers

Within the athletic microsystem, coaches emerged as pivotal ecological actors. When alignment existed, strain was reduced for example: “*[My coach] understands if I miss a session for an assignment.”* (P2-UJ-F-At). This reflects a supportive *Process × Context* interaction. Flexibility reduced identity conflict and preserved academic legitimacy. The proximal process (training) remained intact without undermining the parallel academic process. However, misalignment produced tension in cases were “*sometimes the coach expects me to be there no matter what… it feels like academics don’t matter”* (P3-UJ-F-Ru) or “*You can’t just skip practice… you know the coach expects you to be there”* (P16-NWU-M-Ru). These quotes illustrate ecological competition. The athletic microsystem asserts primacy, delegitimising academic demands. The student-athlete is then forced into identity arbitration, a regulatory task that intensifies psychological load. Thus, coaches functioned not merely as mentors, but as structural gatekeepers influencing how compatible the two systems felt. These narratives highlight the importance of open and effective communication with coaches, where alignment between athletic expectations and academic realities can mitigate stress, whereas misalignment may exacerbate pressure and conflict (23). Such findings resonate with research showing that supportive coach–athlete relationships are critical for fostering not only athletic performance but also academic persistence and psychological well-being (24).

#### 5.2.3 Lecturers and the burden of self-advocacy

The academic microsystem presented a different form of strain. Several participants described the need for repeated explanation “*why I can't attend a class”* (P4-UJ-F-Ru). The repetition embedded in “every time” signals cumulative labour. Academic accommodation was not automatic; it required emotional and communicative effort. In PPCT terms, the proximal process of negotiation became an additional developmental task layered onto study and performance demands. Another participant noted that experiences varied by course, observing that “*lectures differ from lecture to lecture”* (P10-UJ-F-At) or that “*some lecturers understand, but others don't really see sport as a reason”* (P16-NWU-M-Ru). Where lecturers were responsive, strain decreased. Where flexibility depended on personal relationships, uncertainty increased. Institutional support was therefore relationally mediated rather than structurally guaranteed. These experiences align with research highlighting that student-athletes’ ability to manage dual careers is influenced by lecturer awareness, institutional support, and communication (26, 46). Research with Division I student-athletes in the United States of America indicates that perceived academic support from staff, along with academic identity and degree commitment, significantly predicts institutional commitment and reduces intent to withdraw, highlighting the importance of academic integration for student-athletes (47). Furthermore, recent evidence suggests that advancements in blended learning and improved communication between university management, academic staff, and student-athletes can strengthen support structures, fostering greater flexibility, engagement, and academic persistence (48).

#### 5.2.4 Peer systems as emotional regulators

In contrast to the lecturers that sometimes-intensified strain, peer networks frequently operated as emotional stabilisers within the dual-career ecology. Participants emphasised that teammates and fellow student-athletes “*understand the pressure and the tiredness”* (P14-NWU-M-Ru). This shared experiential base reduced the explanatory burden often associated with dual-role strain. Within the PPCT framework, this illustrates how microsystem similarity enhances emotional validation: when peers occupy comparable roles, proximal processes require less justification and more mutual recognition. Teammates, in particular, emerged as co-regulators of motivation. One participant explained that “*my teammates understand the stress… we motivate each other, it really helps”* (P3-UJ-F-At), while another reflected that “*they pushed me to keep going… they are role models in a way”* (P6-UJ-F-Ru). These accounts demonstrate that peer interactions were not merely social exchanges; they were performance-sustaining processes. Through daily training, shared fatigue, and collective goal pursuit, teammates reinforced standards while simultaneously normalising struggle. In PPCT terms, these repeated *Person × Process* interactions within the athletic microsystem strengthened persistence and buffered self-doubt. Motivation was redistributed across the group rather than carried individually. Importantly, peer regulation functioned both emotionally and behaviourally. Emotional validation, “*they get it”,* reduced feelings of isolation, while behavioural modelling, “*they pushed me”,* elevated effort norms. This dual function stabilised identity integration: being surrounded by others who were similarly committed reinforced the legitimacy of the dual-career pathway. Belonging therefore acted as a protective mechanism during periods of overload.

Interactions with non-athlete classmates, however, were more variable, revealing how contextual differences shape proximal process quality. Some academic peers provided instrumental support, such as sharing notes or clarifying missed material. As one participant described, “*My classmates are very supportive… they tell me what I missed out on, or what I need to do”* (P11-UJ-F-Ru). Another similarly noted, “*If I miss class, someone will send me the work or explain what I missed”* (P16-NWU-M-Ru). These forms of assistance illustrate how the academic microsystem can facilitate continuity despite absence. Here, *Person × Context* alignment is evident: when classmates recognise and accommodate dual-career demands, academic strain is reduced and engagement is sustained. Yet this alignment was not universal. Other participants described subtle relational gaps. One reflected that “*sometimes it feels like my classmates don't get it… I'm juggling so much more than them”* (P9-UJ-M-Ho). Another articulated the cumulative weight of competing demands: “*Balancing class schedules, gym, field sessions, physiotherapy, family responsibilities… never a typical day”* (P1-UJ-F-At). These accounts signal microsystem dissonance. Where experiential similarity was absent, participants felt misunderstood or differentiated, reinforcing a sense of separateness within the academic environment. Across Theme 2, what becomes evident is that proximal processes were not neutral routines but emotionally charged sites of regulation. Peer systems either absorbed strain or amplified it, depending on the degree of experiential overlap and contextual understanding. When ecological alignment was present, daily interactions strengthened belonging, validated effort, and protected psychological resources. When misalignment occurred, explanatory labour increased and isolation intensified. Thus, peer networks functioned as critical emotional regulators within the dual-career pathway, shaping whether sustained motivation translated into resilience or erosion. These findings are consistent with research showing that peer networks buffer stress and reinforce identity in athletic contexts (49). Empirical studies indicate that peer support can significantly reduce perceived stress and enhance psychological resilience among university athletes, highlighting the stress-buffering role of supportive peer relationships (50). Teammate connectedness specifically has been associated with greater maintenance of athletic identity and improved mental health, further underscoring the importance of social bonds among peers (51).

### 5.3 Theme 3: institutional structures; support that exists, but does not always hold

Beyond immediate peer and coach relationships, participants reflected on the broader institutional architecture shaping their dual-career engagement. Within the PPCT framework, this theme foregrounds the *Context* dimension. Institutional structures formed the exosystem and mesosystem conditions under which proximal processes unfolded. Yet contextual provision did not automatically stabilise high-intensity engagement. Its impact depended on clarity, accessibility, and alignment with individual characteristics.

#### 5.3.1 Structural support as cognitive and emotional buffer

When institutional systems functioned coherently, participants described a reduction in cognitive load. One student-athlete explained that when the sport office assisted with lecturer communication during absences, “*that support makes everything more manageable”* (P2-UJ-F-At). The phrase “*more manageable”* is significant, institutional mediation did not remove pressure, but it redistributed it. In PPCT terms, coordinated *Context* strengthened proximal processes by smoothing mesosystem interaction between sport and academics. Rather than investing energy in negotiating legitimacy, the student-athlete could direct effort toward performance and learning. Here, *Context* moderated *Person*-driven intensity, making sustained engagement ecologically viable. Similarly, access to integrated facilities and professional staff reinforced perceptions of structural competence. Descriptions of having “*everything we need”* (P18-NWU-F-Ne) or access to multidisciplinary teams suggested environments where development was scaffolded rather than improvised. In these cases, institutional alignment enhanced confidence and reinforced identity coherence.

Supportive sporting environments extended beyond performance outcomes to include emotional well-being and academic persistence. Participants described how being “*surrounded by people who understand what I'm doing and offer the support I need”* (P2-UJ-F-At) or having access to individuals to discuss “*academics or mental health around being an athlete”* (P4-UJ-F-Ru) enhanced their engagement. NWU participants noted that support was present but contingent on awareness and initiative, with one stating, “*there's support if you know how to ask… otherwise you struggle”* (P22-NWU-F-Ho). Conversely, unclear or inconsistent support increased stress, particularly during injury, financial strain, or academic difficulty, as one participant explained: “*not having a support system… losing my scholarship while injured… that was terrible”* (P7-UJ-F-Ru). These accounts indicate that accessibility, clarity, and proactive communication are crucial for institutional support to be effective. Where support was well-structured and staff were engaged, student-athletes reported higher satisfaction and more effective management of dual-career responsibilities (27). In contrast, ambiguous or inconsistent support, or systems that relied on self-advocacy, contributed to stress, uncertainty, and perceived inequities (15, 28, 52).

#### 5.3.2 Conditionality and policy-practice gap

Despite formal provision, many participants described support as interpretively fragile. As one noted, “*support exists, but interpretation matters…”* (P1-UJ-F-At). This statement reveals critical tension: policy presence did not ensure consistent enactment. Others described the burden of self-initiation, explaining that lecturers “*don't help me besides me having to text or email”* (P7-UJ-F-Ru). Support was therefore reactive rather than embedded. The student-athlete became the primary coordinator of accommodation, translating institutional policy into practical resolution on a case-by-case basis. These dynamics highlights a clear *Person × Context* interaction. Access to structural flexibility depended partly on individual assertiveness, communication confidence, and relational navigation skills. Institutional coherence was thus uneven, not structurally absent, but dependent on personal agency. Accommodation frequently hinged on rapport rather than transparent procedure. Participants acknowledged that “*having a good personal relationship with your lecturer helps”* (P9-UJ-F-Ru). While relational goodwill humanised the system, it simultaneously exposed structural vulnerability. When flexibility relies on discretionary benevolence, support becomes inconsistent and potentially inequitable. In ecological terms, mesosystem coordination (sport–academic interface) lacked full institutional integration. The system held, but selectively. This pattern aligns with dual-career literature indicating that institutional policies, when not clearly communicated or consistently enacted, tend to be experienced by student-athletes as fragmented and person-dependent, resulting in uneven support outcomes (15, 24).

#### 5.3.3 Temporal rigidity and the transfer of regulatory burden

Scheduling practices revealed a less visible, but highly consequential, structural tension: institutional time operated as fixed and non-negotiable, while student-athlete time remained elastic and absorbent. Participants repeatedly described adapting to predetermined timetables rather than experiencing coordinated flexibility. As one explained, “*the schedules are in place, and we just work around it”* (P5-UJ-F-Ru). Another described the perpetual vigilance required: “*I'm always going to have to be on my toes”* (P8-UJ-F-At). The phrase work around it signals accommodation without reciprocity. Academic calendars, assessment deadlines, training schedules, and competition fixtures existed as parallel systems, rarely structurally integrated. When clashes occurred, resolution depended on individual adjustment rather than systemic redesign.

Within Bronfenbrenner's PPCT framework, this highlights the salience of the chronosystem. *Time* was not neutral; it functioned as a structuring force shaping the intensity and sequencing of proximal processes. Institutional time remained stable, lectures occurred at fixed hours, assessments followed standardised timelines, and competitions adhered to external governing bodies. In contrast, student-athletes experienced time as compressed, overlapping, and cumulative. This asymmetry produced what can be conceptualised as a transfer of regulatory burden. Rather than *Context* recalibrating to accommodate dual-role intensity, *Person*-level regulation intensified. Students described heightened planning, anticipatory communication, and constant schedule recalculation. The vigilance captured in “*always on my toes”* reflects a sustained cognitive load, an ongoing monitoring of potential conflict between microsystems. Research further suggests that ambiguity around procedures for assessment rescheduling, attendance concessions, and travel-related absences increases uncertainty and stress, particularly when support depends on individual staff discretion rather than shared institutional guidelines (26, 53).

#### 5.3.4 Resource availability vs. resource utilisation

While institutional resources were widely acknowledged, their use was inconsistent. One participant noted, “*Impossible for you to fail if you use all the resources at your disposal”* (P10-UJ-F-At), reflecting strong confidence in structural support and signalling substantial institutional investment. Yet, others reported limited engagement: “*There is support… but for me, none”* (P6-UJ-F-Ru). This divergence highlights that availability alone does not guarantee utilisation; perceived relevance, trust, and alignment with individual needs shape engagement. From a PPCT perspective, effective support requires that contextual provisions resonate with *Person*-level meaning systems to facilitate positive proximal processes. Even when formal support exists, student-athletes may selectively engage with them if they appear misaligned with immediate needs or carry stigma, reinforcing reliance on informal coping strategies (16, 28). Thus, the mere presence of resources is insufficient; integration into the daily rhythms of dual-career life and alignment with personal meaning systems are essential for effective utilisation. Although student-athlete mental health has become an increasing focus in the literature (54), participants reported limited engagement with formal support services, primarily due to time constraints and competing demands. This supports arguments that mental health interventions must be integrated within sport environments rather than positioned as additional, time-consuming services (55). Despite these challenges, many participants expressed generally positive appraisals of their dual-career journeys. This apparent optimism may reflect adaptive coping strategies such as positive self-talk, goal orientation, and growth mindset, which have been associated with enhanced self-confidence and stress regulation in athletic populations (56–58).

### 5.4 Theme 4: time, transition, and the slow accumulation of strain and growth

*Time* emerged not merely as background context, but as a structuring force shaping how motivation, relationships, and institutional conditions unfolded. Within the PPCT framework, the chronosystem captures both developmental timing (across years) and cyclical intensification (within seasons). Across accounts, dual-career engagement was experienced as cumulative, iterative, and temporally uneven. Rather than a linear adjustment process, participants described recurring cycles of destabilisation, recalibration, and consolidation.

#### 5.4.1 Adaptation over time: transition as developmental disruption

Entry into university disrupted previously stabilised routines. Academic expectations were initially opaque, “*you don't know what's expected in first year… you figure it out”* (P1-UJ-F-At), positioning adaptation as emergent rather than guided. The necessity to “*figure it out*” signals an abrupt transfer of regulatory responsibility to the individual. Simultaneously, early adjustment required psychological compromise, as it “*needs to go both ways”* (P20-NWU-M-Cr), indicating that dual-role engagement demanded negotiation rather than dominance of one domain over the other. The cumulative effect was destabilising: “*first year was hard… there was a lot to adjust to”* (P21-NWU-M-Cr). These reflections frame early maladjustment as developmental recalibration rather than personal inadequacy. The transition altered *Context* (greater autonomy, intensified performance demands) and *Process* (denser, less scaffolded proximal interactions), temporarily weakening regulatory equilibrium. Over time, however, prior exposure became regulatory capital. Having “*done it before and succeeded” generated confidence in the ability “to excel in both”* (P2-UJ-F-At), illustrating how earlier chronosystem experiences strengthened Person-level efficacy. Anticipatory planning reduced uncertainty, “*now I plan my week… there's less stress because I know what to expect”* (P22-NWU-M-Cr), not because demands diminished, but because they became predictable. Mastery accumulated through repetition. Yet adjustment remained cyclical. Each academic year reintroduced congestion, requiring renewed recalibration. Time repeatedly restructured demand rather than resolving it.

Collectively, these reflections indicate an iterative adaptation process, in which student-athletes draw on prior experiences to refine self-regulation and progressively develop strategies for managing academic and athletic demands (44, 59). However, the start of each academic year consistently emerged as a renewed adjustment period, requiring reorientation to academic schedules, institutional processes, and concurrent peak competition demands. This cyclical pattern mirrors findings from higher education and dual-career research, which identify transitional phases as periods of heightened strain and recalibration rather than linear progression (60, 61). These recurring adjustments underscore the temporal dimension central to the PPCT model, illustrating how *Time* operates not merely as duration but as a dynamic force that periodically intensifies demands and disrupts equilibrium (38).

#### 5.4.2 Critical periods and temporal intensification

Pressure intensified sharply when academic and athletic calendars converged. Temporal compression became evident when “*[training] camp fell during final exams”* (P3-UJ-F-Ru), collapsing recovery time and amplifying cognitive load. Similar calendar conflicts have been widely documented in dual-career research, where academic assessments frequently coincide with major competitive periods, creating concentrated workload peaks for student-athletes (62). Mobility demands further fragmented learning environments, as one participant noted: “*difficult to learn on the bus… right at the test on a Monday”* (P12-NWU-F-Ho), demonstrating how travel associated with sport participation can disrupt study routines and academic engagement (63). During peak preparation phases, training demands also intensified, with one participant explaining that “*for two weeks before USSA, we train six hours a day”* (P15-NWU-M-Ho). Such intensive preparation periods have been linked to increased psychological fatigue when combined with academic obligations (40). These experiences illustrate chronosystem collisions, where independently operating institutional systems intersect to create concentrated, cyclical strain rather than constant overload. Dual-career research similarly highlights that student-athletes often experience clustered workload peaks as sport and academic demands overlap (41). From a PPCT perspective, these patterns reinforce that the timing, sequencing, and overlap of environmental demands, rather than their presence alone, significantly shape proximal processes and developmental outcomes (38).

#### 5.4.3 Cumulative fatigue and long-term strain

Beyond acute peaks, a more chronic accumulation emerged. “*Fatigue. Being tired… each and every single day… brain is overstimulated”* (P7-UJ-M-Ru) conveys sustained cognitive and emotional saturation rather than episodic stress. Daily schedules offered little restorative space “*you have a full day and you can't rest in between”* (P12-NWU-F-Ho), producing a continuous sense of pursuit captured in the repetition: “*catch up, catch up, catch up”* (P14-NWU-F-Ho). The language reflects temporal deficit, where demands persistently outpaced recovery. Dual-career engagement therefore required ongoing cognitive switching and emotional regulation. Recovery was partial and often deferred, allowing strain to layer over time. When disruption occurred in one domain, its effects reverberated. Injury destabilised academic engagement, having “*started slacking on my academics, failed quite a few modules”* (P7-UJ-M-Ru), demonstrating cross-system interdependence. Deselection triggered identity disturbance, becoming “*really, really, really sad… considering quitting”* (P6-UJ-M-Ru). These episodes expose the fragility underlying sustained high performance. In PPCT terms, proximal processes across microsystems are interlinked; disturbance in one weakens stability in others. *Time* deepened these consequences by allowing strain to accumulate (64).

### 5.5 Developmental consolidation and reflective growth over time

Accumulated exposure to the demands of dual-career participation did not exclusively produce strain; it also facilitated developmental growth and consolidation. Participants described how adaptation emerged gradually through prolonged engagement with the academic–athletic environment. Competence and adjustment required time, with one athlete explaining that “*it took me about already three years to do all that”* (P7-UJ-M-Ru). Such reflections suggest that successful navigation of dual-career demands was not immediate but developed through repeated engagement with challenging proximal processes. Over time, these experiences contributed not only to athletic competence but also to broader personal development. One participant reflected that participation had helped them “*not just grow as a player, but as a person… shaping me for the past four years”* (P18-NWU-F-Ne), illustrating how extended exposure to dual-career contexts influenced evolving *Person* characteristics.

Repeated encounters with pressure also fostered increasing metacognitive awareness and adaptive regulation. Participants described learning how to manage demanding situations more effectively through experience. One athlete noted developing strategies for “*how to respond to pressure”* (P2-UJ-F-At), while another reflected that later challenges felt more manageable because “*it's like I've been through this”* (P15-NWU-M-Ho). Prior destabilising experiences thus became resources for future adaptation, allowing athletes to reinterpret strain as informational feedback rather than solely as a barrier. Research on talent development similarly suggests that exposure to challenging environments can support the development of resilience, self-regulation, and psychological skills when athletes are able to reflect on and learn from these experiences (65).

Over time, participants also described a shift in how they interpreted the sacrifices associated with pursuing dual careers. Rather than framing competing demands as externally imposed burdens, many athletes increasingly emphasised personal agency and choice. One participant explained that dual engagement was “*the life I chose… the complaining part doesn't make sense”* (P5-UJ-M-Ru), reflecting a reframing of commitment as a personally meaningful pathway. Even when goals were not fully realised, experiences were frequently interpreted as contributing to broader developmental growth. As one athlete noted, “*even though I wasn't getting what I wanted… I was still growing… mindset, character”* (P10-UJ-M-Ru), while another acknowledged being “*still learning how to navigate failure and rejection”* (P14-NWU-F-Ho). These reflections demonstrate how identity, resilience, and agency evolved through sustained participation in the dual-career environment. Development therefore emerged not only from isolated events but from the cumulative effects of repeated experiences across time. Similar patterns have been identified in athlete development research, where learning, adaptation, and identity formation are understood as iterative processes shaped by ongoing engagement with sport and educational contexts (44, 59). From a PPCT perspective, these findings highlight the significance of the chronosystem, demonstrating how developmental outcomes emerge through the interaction of personal characteristics, environmental demands, and repeated proximal processes unfolding over time (38).

## 6 Limitations and future research

While this study provides valuable insight into how student-athletes navigate dual academic and athletic demands, several limitations should be acknowledged, which also point to important directions for future research. First, the study employed a qualitative, cross-sectional design with a relatively small sample drawn from two HEIs. Although this approach was appropriate for exploring lived experiences in depth, the findings are not intended to be generalisable across all institutional contexts or sporting codes. Future research could build on these insights through larger-scale quantitative or mixed-methods studies to examine the prevalence and strength of the identified patterns, particularly those related to burnout, self-regulation, and institutional engagement. Second, data relied on participants’ retrospective self-reports, which may be subject to recall bias or social desirability effects, especially in relation to coping, resilience, and perceptions of support. Longitudinal designs are recommended to capture how student-athletes’ experiences, coping strategies, and interactions with academic and sport systems evolve over time. Such approaches would be particularly valuable in examining chronosystem influences, including transition into university, peak competition periods, injury, and progression through academic programmes. Third, the study foregrounded student-athletes’ perspectives and did not include the views of lecturers, coaches, academic advisors, or support staff. While this focus aligns with the study's narrative and bioecological orientation, future research should adopt multi-perspective ecological designs to better understand how institutional policies are interpreted, enacted, and negotiated across different system levels. Including these stakeholders may help identify structural inconsistencies and points of misalignment between policy intent and lived practice. Fourth, although participants were drawn from two institutions, the study did not aim to conduct a formal institutional comparison. As a result, contextual differences in organisational culture, informal practices, and resource coordination may not have been fully captured. Future research could adopt comparative case-study designs to explore how institutional logics, governance structures, and support models shape student-athletes’ engagement with academic and athletic demands across different settings. Finally, while the findings suggest that burnout, emotional regulation, and fatigue may spill over between athletic and academic domains, these relationships were not measured directly. Future studies should examine these mechanisms more explicitly, incorporating variables such as sleep quality, recovery practices, and psychological skills use. Investigating the effectiveness of embedded interventions, such as integrated mental health support, structured self-advocacy training, and coordinated academic flexibility frameworks, would further contribute to evidence-based approaches that reduce reliance on individual resilience alone.

## 7 Practical implications

**Figure 1:**
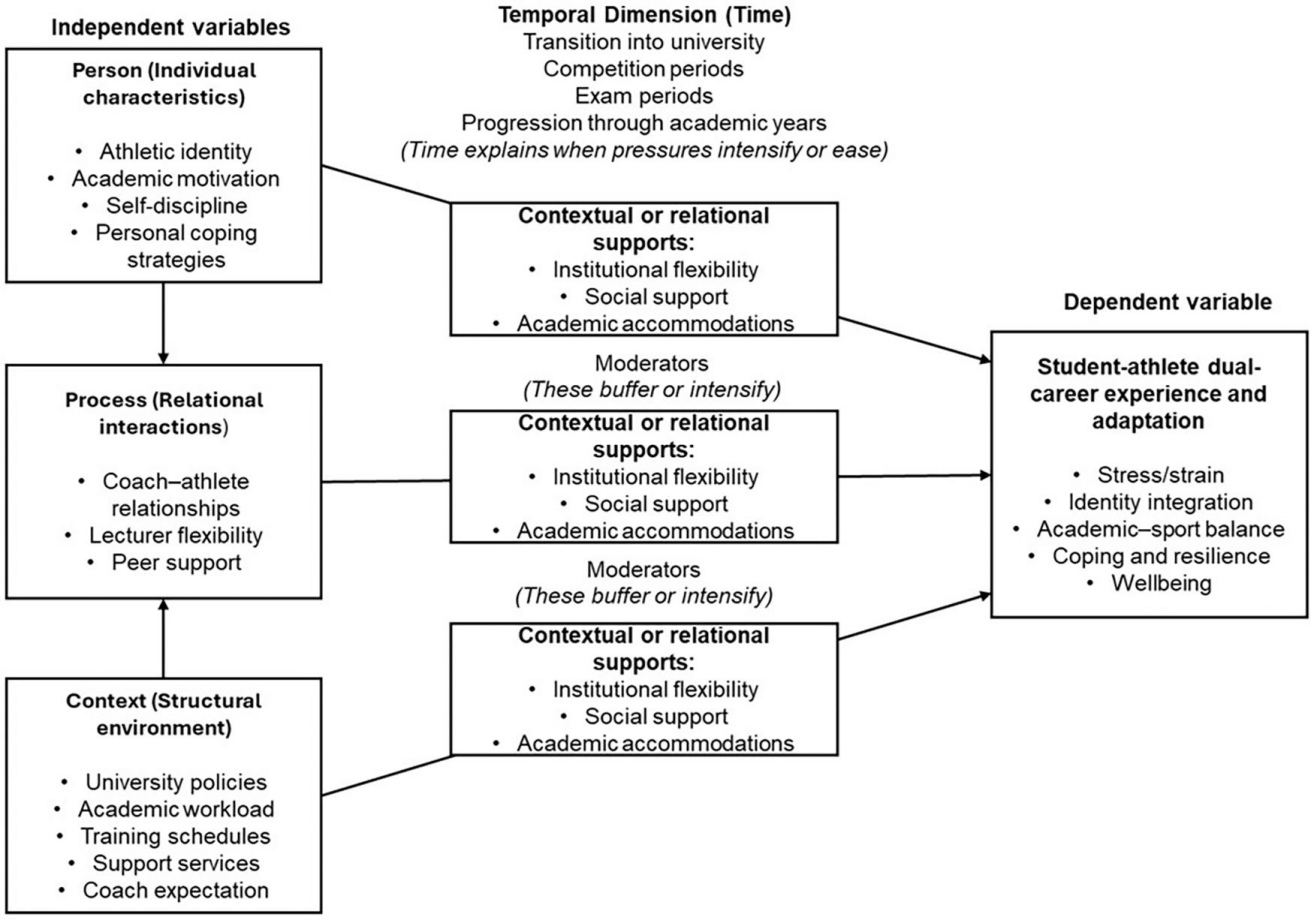
Dual-Career engagement framework.

The findings of this study offer several practical implications for HEIs, sport departments, and academic staff involved in supporting student-athletes. Grounded in a bioecological perspective and operationalised through the Dual-Career Engagement Framework (Figure 1), these implications emphasise that effective dual-career support extends beyond the mere presence of structures and resources, requiring attention to how student-athletes engage with and navigate these environments over time.

At the personal (*Person*) level, the prominence of dispositions such as motivation, self-discipline, resilience, and emotional regulation suggests that dual-career support strategies should actively cultivate these capacities rather than assume they are pre-existing or evenly distributed. While these traits enabled persistence and high performance, they also heightened vulnerability to burnout and psychological strain. Institutions can therefore develop structured skills programmes that target adaptive coping, self-regulation, boundary setting, and self-compassion. Embedding these interventions directly within sport environments, rather than as optional or external services, may improve accessibility and reduce the stigma associated with mental health support. Proximal processes (*Process*), particularly self-advocacy and interpersonal interactions, were identified as critical mechanisms through which student-athletes negotiate academic and athletic demands. While self-advocacy fosters autonomy and confidence, over-reliance on individual negotiation risks inequity and cumulative stress. HEIs should develop clear, consistently applied academic guidelines that account for temporal realities of training, competition, and recovery. Complementary programmes, such as coach–academic liaison initiatives, peer mentoring, and facilitated study groups, can strengthen positive proximal interactions, ensuring that support is relationally embedded rather than *ad hoc*. At the contextual (*Context*) level, findings emphasise that social and institutional structures are not universally protective; their effectiveness depends on alignment with student-athletes’ lived experiences. While team-based social support was highly valued, it often masked broader social narrowing, leaving academic and family networks underutilised. Universities can encourage diversified social integration by fostering engagement in academic cohorts, interdisciplinary learning opportunities, and peer support networks outside sport. Similarly, structural resources such as facilities, scheduling, travel, and nutrition must be evaluated not only for availability but for usability within daily routines. Coordinated collaboration between sport services, academic departments, and student support units can reduce misalignment, optimise recovery practices, and enhance holistic well-being. Finally, temporal considerations (*Time)* are fundamental. Stress and adaptation are not static; critical periods such as the start of academic terms, peak competition phases, examinations, and injury events repeatedly disrupt balance. Institutions should implement anticipatory planning, including flexible assessment timelines, embedded mental health monitoring, psychological check-ins, and recovery-focused programming, to support student-athletes across these high-demand periods. By attending to the interplay of *Person × Process × Context × Time*, universities can move beyond reactive problem-solving to systemic, ecologically informed strategies that enhance dual-career sustainability.

## 8 Conclusion

This study advances understanding of dual-career engagement by conceptualising student-athlete experiences through Bronfenbrenner's Bioecological PPCT model, demonstrating that success and strain emerge from the dynamic interplay of personal dispositions, relational processes, institutional contexts, and temporal dynamics. Rather than identifying linear determinants, the findings reveal that dual-career outcomes are emergent properties of complex system interactions, where adaptive capacities, proximal processes, and structural supports converge or diverge in shaping well-being and performance.

At a theoretical level, this work highlights the necessity of integrative, systems-oriented frameworks in dual-career research. Personal attributes such as motivation, self-regulation, and resilience interact with proximal processes, including coaching, team cohesion, and self-advocacy, and are further conditioned by institutional flexibility and temporal transitions (21, 38, 42, 66). These interactions elucidate why dual-career stress cannot be fully explained by individual characteristics or structural resources alone: outcomes depend on the quality, timing, and alignment of these interactions, reinforcing ecological perspectives on human development (24, 67). Practically, the findings highlight that institutional coherence and integration are central to sustainable dual-career pathways. Academic accommodations, mental health services, and structural resources are most effective when embedded within the rhythm of athletes’ lived experiences, rather than offered as discrete or *ad hoc* interventions (27, 53, 55, 72). Social support, while protective within the athletic microsystem, must be broadened across academic and personal networks to mitigate hidden dimensions of burnout and facilitate holistic development (61, 68, 69–71). Temporal dynamics further highlight that adaptation is iterative: transitions, peak competition periods, and critical academic phases repeatedly recalibrate the balance between stress and coping, illustrating the centrality of the chronosystem in dual-career trajectories. Conceptually, this study proposes a comprehensive PPCT-informed framework for Dual-Career Engagement (as proposed in Figure 1), positioning a holistic student-athlete experience (including reduced stress/strain and overall wellbeing among others) as the emergent outcome of recursive interactions between *Person, Process, Context,* and *Time*. By further testing this framework, South African HEIs can explore how multiple interacting factors shape student-athlete development, rather than treating issues in isolation. Investigating how personal capacities, relational processes, institutional structures, and timing influence outcomes allows universities to design support that is flexible, contextually relevant, and evidence-informed. Such an approach provides a foundation for moving beyond reactive interventions, helping institutions develop sustainable dual-career pathways that enhance both performance and holistic development.

## Data Availability

The raw data cannot be made publicly available as these would include transcripts of conversations with students and the ethical clearance will not permit it. Requests to access the datasets should be directed to the corresponding author.
